# Bone Scans in Preoperative Investigations of Breast Cancer Cases

**DOI:** 10.7759/cureus.50499

**Published:** 2023-12-14

**Authors:** Islam Mansy, Abdelfatah M Elsenosy, Eslam Hassan, Mujtaba Abdelgader

**Affiliations:** 1 General Surgery and Surgical Oncology, Maadi Armed Forces Medical Complex, Cairo, EGY; 2 Trauma and Orthopaedics, University Hospitals Dorset, Poole, GBR; 3 Trauma and Orthopaedics, Poole General Hospital, Poole, GBR; 4 General Surgery, Cairo University, Cairo, EGY

**Keywords:** bone scintigraphy, metastasis, breast cancer, preoperative, bone scan

## Abstract

Background: Breast cancer constitutes about 28% of all new cancer diagnoses in women, making it the most frequently diagnosed cancer among them. Our objective was to assess the role of bone scans (BS) in preoperative investigations of breast cancer.

Methods: This study involved 105 patients with varying stages of breast cancer, ranging from T1 to T4. We categorized them into three groups: group 1 comprised 40 women with breast cancer who underwent retrospective BS, group 2 included 30 patients with breast cancer who prospectively did not require BS for all cases, and group 3 consisted of 35 women retrospectively diagnosed with breast cancer who did not necessitate BS for all cases. The diagnosis of bone metastasis was confirmed upon obtaining a positive result through bone scintigraphy, subsequently affirmed by another imaging technique such as CT, X-ray, or MRI.

Results: The hospital costs were significantly lower in groups 2 and 3 compared to that of group 1, indicating that performing a BS for every case is unnecessary. It was observed that the time taken for surgery was notably shorter in groups 2 and 3 compared to that of group 1. BS in cases classified as M stage were deemed both costly and time-consuming.

Conclusions: Routine BS are not cost-effective and represent an unnecessary investment of time. They should not be deemed mandatory as preoperative investigations in breast cancer cases. Instead, they should be considered in conjunction with MRI, particularly in cases of T4 breast cancer.

## Introduction

Breast cancer is the most commonly diagnosed cancer in women, accounting for approximately 28% of all new cancer diagnoses, excluding nonmelanoma skin cancers [1]. At diagnosis, the stage of breast cancer is a reliable prognostic indicator [1]. Early-stage diagnosis leads to more favorable outcomes. Axillary dissection or sentinel node biopsy is considered essential in breast cancer management for axillary staging [2]. However, there is ongoing debate about the necessity of conducting entire systemic staging studies for apparently early breast cancers (EBC) [2].

The bone is the primary site for the metastatic development of breast cancer, often identified by osteolytic lesions. The reason for this predilection remains unclear [3]. Imaging is crucial for diagnosing bone metastasis in breast cancer, with bone scintigraphy (BS) being the most frequently used modality [4]. Despite its prevalence, this technique lacks specificity and possesses certain drawbacks.

Bone scans (BS) employ technetium-99m-labeled diphosphonate to detect developing bone formation, advantageous in the early detection of bone metastasis linked to osteolytic metastases [4]. Recent retrospective investigations have cast doubt on the clinical efficacy of BS as a staging technique in breast cancer [5,6]. Previous studies documented favorable outcomes in asymptomatic breast cancer cases, but subsequent investigations failed to replicate these significant results [5,6]. Particularly, when patients have undergone staging computed tomography (CT) scans, questions arise regarding cost-effectiveness and utility [7].

Studies indicate a lower likelihood of bone metastases during staging investigations in patients with breast cancer stage I or II [7,8]. This raises questions about the necessity of routine BS in monitoring such patients, particularly concerning cost-effective healthcare [9].

Due to the high cost of BS and the common occurrence of early-stage breast cancers, substantial healthcare savings could be attained by minimizing or excluding BS for asymptomatic individuals [10].

In our center, a leading facility in Egypt for breast cancer treatment, our tertiary breast clinic previously ordered BS for all breast cancer cases as part of the preoperative metastatic workup. However, guidelines from NICE and NCCN suggest that BS are not mandatory for all cases, recommending consideration only for T4 cases or cases with symptomatic back pain [11]. This practice resulted in increased hospital costs, patient burden, resource consumption, radioactive material exposure, and extended waiting times before surgery.

The primary goal of our study was to evaluate the role of BS in preoperative investigations of breast cancer cases.

## Materials and methods

This study was conducted at the General Surgery & Surgical Oncology Department at Maadi Armed Forces Medical Complex and was approved by the research ethics committee. The data collection began in September 2022, focusing on outpatient clinic records of patients diagnosed with breast cancer since April 2022.

Inclusion criteria

Patients eligible for inclusion in this study were those diagnosed with breast cancer between April 2022 and November 2022 at the General Surgery & Surgical Oncology Department at Maadi Armed Forces Medical Complex. It is imperative that complete clinicopathological data are available for analysis. Additionally, patients across varying cancer stages, ranging from T1 to T4 classifications, were considered for inclusion. Only patients who had provided their consent to participate in the study were included.

Exclusion criteria

Patients who were not considered for this study were those without a confirmed diagnosis of breast cancer. Additionally, individuals with incomplete or insufficient clinicopathological data were excluded. Patients with a history of bone metastasis prior to the study period were also excluded. Finally, patients who did not consent to participate in the study were not included in the analysis.

This encompassed 105 cases, ranging from T1 to T4. Initially, data included the number of cases that underwent BS and the results of abnormal scans. Subsequently, revised standards were implemented, restricting the use of BS to T4 cases and symptomatic bony metastases cases, wherein BS or MRI was considered. The first cycle, spanning until November 2022, involved counting the BS ordered during this period and assessing their outcomes. A retrospective re-audit in January 2023 examined patients diagnosed with breast cancer in November 2022 to ensure the continued application of these changes.

Audit cycle

Problem

Mandating BS as a preoperative assessment for all breast cancer cases resulted in numerous normal scans, leading to financial strain on the hospital, prolonged patient waiting times, and unnecessary radioisotope exposure.

Standard

According to NICE and NCCN Guidelines 2022, BS were recommended only for patients exhibiting signs or symptoms of metastatic disease and those with locally advanced breast cancer.

Data Collection and Analysis

Over 100 cases of breast cancer (ranging from T1 to T4) were reviewed, all with normal bone scan results.

Implementation

BS were excluded from routine preoperative investigations, reserved only for cases displaying signs of metastatic cancer or classified as T4.

Re-audit

Data were collected to confirm the sustained adherence to these changes.

Presentation

The audit findings were presented in a departmental meeting. This study involved 108 patients with varying stages of breast cancer, ranging from T1 to T4. We categorized them into three groups: Group 1 comprised retrospective data from 40 women who underwent BS, Group 2 included prospective data from 30 patients without the requirement for BS, and Group 3 consisted of 35 women retrospectively who did not need BS for their cases.

Diagnosis of Bone Metastasis

A diagnosis of bone metastasis was confirmed when bone scintigraphy showed positive results, further validated by additional imaging modalities, such as CT, X-ray, or MRI [12]. In cases without confirmed bone metastasis, patients underwent biopsy, enrollment in follow-up studies, or both. Patients showing positive findings in alternative imaging despite negative bone scan results were categorized as having bone metastasis. The study found the sensitivity and specificity of bone scintigraphy to be 96% and 97%, respectively. These percentages may be relatively high due to the diagnoses made by experienced nuclear medicine specialists.

Basic clinicopathological data and results of all BS were documented. Both the bone scan results and subsequent evaluations based on those findings were recorded. Metastases detected directly from the bone scan or through follow-up studies prompted by the scan's findings were considered positive. Benign or normal results from the bone scan were treated as negative, while indeterminate findings were considered false positives if further testing or observation didn't confirm the presence of metastases.

Statistical analysis

The statistical analysis was performed using Statistical Product and Service Solutions (SPSS) v28 (IBM Inc., Armonk, NY). Quantitative variables were expressed as standard deviation (SD) and mean, and a comparison between the two groups was done using the ANOVA (F) test. Qualitative variables, presented as percentages (%) and frequencies, were analyzed using the chi-square test. A two-tailed p-value of <0.05 was considered statistically significant.

## Results

Table 1 shows that there were insignificant differences among the studied groups regarding the baseline characteristics (age and residence).

**Table 1:** Baseline characteristics of the studied patients Data are presented as mean ± SD or frequency (%).

		Group 1 (n=40)	Group 2 (n=30)	Group 3 (n=35)	P value
Age (years)		45.28 ± 9.19	46.97 ± 8.38	44.49 ± 9.66	0.542
Residence	Urban	22 (55%)	19 (63.3%)	23 (65.7%)	0.606
	Rural	18 (45%)	11 (36.7%)	12 (34.3%)	

Menstruation cycle and estrogen receptor status were insignificantly different among the studied groups (Table 2).

**Table 2:** Menstruation cycle and estrogen receptor status of the studied patients Data are presented as frequency (%).

		Group 1 (n=40)	Group 2 (n=30)	Group 3 (n=35)	P value
Menstruation cycle	Premenopausal	17 (42.5%)	14 (46.7%)	17 (48.6%)	0.932
	Postmenopausal	23 (57.5%)	16 (53.3%)	18 (51.4%)	
Estrogen receptor status	Positive	18 (45%)	20 (66.7%)	22 (62.9%)	0.136
	Negative	22 (55%)	10 (33.3%)	13 (37.1%)	

Table 3 shows that there were insignificant differences among the studied groups regarding the tumor stage (T1, T2, T3, and T4).

**Table 3:** Tumor stage of the studied patients Data are presented as frequency (%).

	Group 1 (n=40)	Group 2 (n=30)	Group 3 (n=35)	P value
T1	20 (50%)	13 (43.3%)	14 (40%)	0.063
T2	5 (12.5%)	8 (26.7%)	6 (17.1%)	0.308
T3	9 (22.5%)	5 (16.7%)	9 (25.7%)	0.675
T4	6 (15%)	4 (13.3%)	6 (17.1%)	0.912

M stage (M1, M0) was insignificantly different among the studied groups as shown in Table 4.

**Table 4:** M stage of the studied patients Data are presented as frequency (%).

	Group 1 (n=40)	Group 2 (n=30)	Group 3 (n=35)	P value
M1	4 (10%)	2 (6.7%)	6 (17.1%)	0.390
M0	36 (90%)	28 (93.3%)	29 (82.9%)	

BS results

In the initial cycle, 40 BS were ordered for 40 patients. Among these scans, only four revealed abnormalities. Subsequent MRI examinations of these abnormal BS indicated that these changes were related to old spine fractures, disproving bone metastases. To conclude the first cycle of this audit, prospective data were collected from the outpatient clinic. Between September 2022 and November 2022, following standard guidelines, only six out of 30 breast cancer patients had BS ordered, all of which were for T4 cases. The results of these six BS were normal. Additionally, the analysis revealed that, out of 35 breast cancer patients, only six with T4 breast cancer had BS ordered, all of which showed normal results.

Influence of BS on Hospital Cost and Operation Time

Comparing groups 2 and 3 to group 1, the cost of hospitalization was significantly lower, affirming that BS are not necessary for all cases. They should be reserved for cases diagnosed with T4 or experiencing severe back pain. Furthermore, the time taken for surgeries was notably faster in groups 2 and 3 compared to that in group 1. Conducting BS in M-stage cases was found to be both costly and time-consuming.

## Discussion

The occurrence of distant metastases in recently diagnosed breast cancer patients is rare initially, yet they can emerge within months after the initial staging [13]. Previous research involving 466 patients diagnosed with primary breast cancer documented that 4.8% developed distant metastases. Literature indicates that the most common sites for these metastases are the bones, with an incidence rate ranging from 1.4% to 6.8%, followed by the liver (0.6%-2.6%) and the lungs (0.4%-3.7%) [14].

In a retrospective analysis by Berclaz et al. involving 266 patients, 8.6% were found to have distant metastases at the time of diagnosis [15]. Their analysis highlighted an exclusive occurrence of distant spread at the T2 stage and in cases involving axillary lymph node involvement. Consequently, they proposed discontinuing certain staging procedures in asymptomatic patients with negative axillary lymph nodes in line with Bares' recommendations [16]. However, Müller et al.'s study did not validate these findings. They observed distant metastases in four patients without positive axillary lymph nodes, with metastases even detected at the T1c stage [14].

Considering that, during follow-up, 25% of patients without nodal involvement develop distant metastases and 30% of these cases result in death due to metastatic disease, it becomes imperative to explore alternative approaches to managing such cases.

Regarding the BS results, the first cycle showed that out of 40 patients, 40 BS was ordered. While interpreting the results of these 40 BS, we found that only four BS showed abnormal results. MRI was done for these abnormal BS, and it showed that these changes were all related to old spine fractures. Bone metastases were disproved. Out of 30 patients diagnosed with breast cancer, only six BS were ordered. All six BS were ordered to T4 breast cancer cases. The results of the six BS were normal. Data analysis showed that, out of 35 patients diagnosed with breast cancer, only six BS were ordered to cases with T4 breast cancer. The result of BS was normal.

Based on the finding that early-stage breast cancer staging by bone scintigraphy may not be cost-effective, all new breast cancer patients at our institution undergo a baseline bone scintigraphy. The cost of the hospital was significantly lower in groups 2 and 3 compared to that of group 1 that ensures that BS is not necessary for all cases, and we have to do it in the cases diagnosed with T4 or had severe back pain. Additionally, the time to the surgery became faster in groups 2 and 3 compared that of to group 1. BS in M stage was a cost and time wasted.

In 1995, around 182,000 cases of breast cancer were estimated in females. Among these, 55%, or roughly 100,100 cases, were confined solely to the breast. A projected cost of $246,246,000 would be incurred if yearly BS were conducted over a five-year follow-up period, assuming a 30% recurrence rate during this time (equivalent to 6% annually) [17,18]. This estimation is based on a cost of $600 for a comprehensive body BS. Further refinement of false-positive testing through follow-up studies would lead to added expenses. It is anticipated that 41,041 cases of false-positive results could occur annually, considering a 10% false-positive rate. Estimating the cost of evaluating false-positive tests is challenging but likely substantial.

An alternative view on the expense of bone scintigraphy as a screening tool involves calculating the cost required to detect recurrent disease cases [19]. Thomsen et al. [20] conducted a study where they discovered that it took 234 BS to identify a single case of bone metastases. These scans were conducted semiannually in the first year and then annually thereafter. According to their findings, the cost of identifying one instance of recurring disease would total $140,400. Therefore, utilizing bone scintigraphy as a screening method for detecting recurring disease in early-stage breast cancer patients results in significant healthcare costs [5].

At the time of diagnosis, only 3% of T1-2N0 patients had distant metastases, compared to 30% of T3-4 or N2 patients, according to Samant and Ganguly [21].

In their research, Gerber et al. [22] found that patients with larger tumor sizes (pT≤2.0 cm: 1.6%, pT 2.1-5.0 cm: 3.0%, pT>5 cm: 15.1%) and lymph node involvement (pN0: 1.9%, pN1-3: 1.8%, pN4-9: 4.0%, pN≥10: 18.7%) had a higher likelihood of experiencing distant metastases. Additionally, the authors highlighted that patients without lymph node involvement and those with one to three affected lymph nodes shared a similar risk of developing initial metastatic disease (1.9 vs. 1.8%). They suggest regular staging tests for individuals at elevated risk to reduce the necessity for unnecessary investigations and expenses.

Several studies have explored the use of BS in the early stages of breast cancer, yielding varied results. Schaffer et al. [23] and Butzelaar et al. [24] concluded that conducting preoperative scans for women with minimal and preclinical breast cancer was unnecessary. Butzelaar et al. [24] opted against routine preoperative scanning for patients with stage I (T1, T2, N0, N1a) breast carcinomas, as only 3.4% (3 out of 90) showed positive results. Baker et al. [25] similarly deemed them not beneficial in clinical stages I and II. However, Charkes et al. [26] recommended preoperative BS for all candidates undergoing radical mastectomy, citing that hidden bone metastases partly contributed to the failure of radical mastectomy in curing breast cancer patients. Gerber et al. [27] emphasized that, while initial yields from preoperative scans were low, their combination with sequential postoperative scans served as a sensitive indicator of developing metastatic disease after mastectomy [28]. Blair [29] reviewed BS of 92 patients with stages I and II breast cancer and found a correlation between normal scans and a more positive outlook regarding recurrence and survival. However, Blair's data did not demonstrate that early detection and treatment of bone metastases improved prognosis, casting doubt on the ultimate value of such scans.

Research conducted by Kunkler et al. [30] and Butzelaar et al. [24] reached the consensus that employing standard BS as a screening measure for detecting metastases in recently diagnosed asymptomatic individuals with early stages of breast cancer lacks validation. These studies found that skeletal scintigraphy abnormalities were observed in merely 3%-7% of patients with stage I-II breast cancer. Moreover, the abnormal results from BS could not be confirmed as actual metastases, and the rate of false positives reached as high as 13.6%.

## Conclusions

In conclusion, our study underscores a pivotal shift in the approach to preoperative investigations for breast cancer cases. By adhering to stringent guidelines advocating targeted use, rather than routine application, we have unveiled a significant reduction in unnecessary BS. Our findings unequivocally support the assertion that mandatory BS for all breast cancer patients are superfluous, financially burdensome for hospitals, time-consuming for patients, and devoid of substantial clinical benefit. Instead, our results advocate for a selective approach, recommending BS solely in cases of T4 breast cancer or when patients present with symptomatic indications of metastatic bone disease.

The implications of this study are far-reaching, proposing a paradigm shift in clinical practice. The evidence presented strongly advocates for revising the current standard of care, emphasizing the judicious and targeted use of BS. By aligning with established guidelines, hospitals can curtail unnecessary healthcare expenses, expedite preoperative processes, and, importantly, spare patients from undue exposure to radioisotope without significant diagnostic yield. This tailored approach, supported by our data, not only streamlines healthcare delivery but also ensures efficient resource allocation, optimizing patient care and contributing to a more sustainable healthcare system.
